# Invasion of old world *Tamarix chinensis* and *T. ramosissima* in the new world: ecological niche shifts during the invasion process

**DOI:** 10.1186/s12870-026-08415-y

**Published:** 2026-03-02

**Authors:** Zhuowen Wang, Xiang Shi, Lele Liu, Weihua Guo

**Affiliations:** 1 College of Urban and Environmental Sciences, Shihezi University, No. 82 North 4th Road, Shihezi, 832003 Xinjiang China; 2 School of Life Sciences, Shandong University, 72 Binhai Road, Qingdao, 266237 China

**Keywords:** Climate change, Ecological niche, Ecological niche model, Migration, Niche analysis, Plant invasion

## Abstract

Biological invasions under global climate change can profoundly affect ecosystem functions and biodiversity. Understanding how invasive species adjust their ecological niches in novel environments is essential for predicting invasion risks and informing management strategies. This study focuses on two rapidly expanding *Tamarix* species in North America, *T. chinensis* and *T. ramosissima*, aiming to determine whether niche shifts occurred during invasion and to identify their potential drivers. We initially considered 19 bioclimatic variables for analysis, but retained eight after correlation filtering. These variables were then used to compare populations between the native range (Eurasia) and the invaded range (North America) to identify the key climatic determinants of distribution patterns. Niche overlap analyses revealed low niche similarity between Eurasian and North American populations, while niche centroid shifts indicated a tendency of the invaded populations toward drier and warmer environments, accompanied by a slight contraction trend. Using the MaxEnt model with current climate data (1970–2000) and future projections for 2070 under SSP1-2.6 and SSP5-8.5, we found that suitable habitats for both species are expected to expand further across the Northern Hemisphere and shift markedly toward higher latitudes. These findings provide a novel empirical perspective for exploring the ecological niche adaptation of invasive species and their responses to environmental changes.

**Supplementary Information:**

The online version contains supplementary material available at 10.1186/s12870-026-08415-y.

## Introduction

Global climate change and biological invasions are increasingly impacting biodiversity, ecosystem functioning, and the use of regional resources as well as socio-economic activities [1]. Invasive species often exhibit strong environmental adaptability and high reproductive capacity, and in introduced regions they frequently lack co-evolved natural enemies, enabling them to rapidly expand and occupy new ecological niches [2]. This expansion not only leads to habitat loss and declines of native species, but also alters hydrological regimes, nutrient cycling, and community structure, resulting in long-term and often irreversible ecosystem degradation [3]. Therefore, understanding the ecological and evolutionary mechanisms underlying successful invasions, particularly whether niche shifts occur during the invasion process, is crucial for assessing invasion risks and formulating effective management strategies. Ecological niche models (ENMs), which statistically quantify the relationships between species distributions and environmental factors, have become powerful tools for predicting invasion potential and guiding management [4, 5]. Many ENM-based studies rely on the assumption of niche conservatism; however, accumulating evidence suggests that this assumption is not universally valid. For instance, Asian carp and *Spartina alterniflora* have been shown to exhibit significant niche shifts in invaded regions, potentially driven by adaptive evolution or enemy release [6, 7]. Despite the growing body of research on invasive species niche dynamics, comprehensive comparative analyses between native and invaded ranges remain limited, particularly for closely related taxa. Addressing this gap is essential for determining whether such taxa exhibit similar or divergent niche dynamics during invasion. This study focuses on two representative invasive *Tamarix* species—*Tamarix chinensis* and *T. ramosissima*. These species are native to Eurasia and were introduced to the United States and South Africa in the early 20th century for windbreaks, soil stabilization, and landscape greening [8, 9]. In the introduced range, however, they have rapidly expanded and become highly invasive. Early remote-sensing assessments indicated that *Tamarix* had already invaded roughly 600,000 ha in the western United States [10]. More recent modelling work using updated occurrence data further suggests that its potential suitable habitat is much larger than previously estimated [11]. *Tamarix* invasions have significant ecological and resource management impacts, including increased soil salinization, altered fire frequency and intensity, exacerbated water scarcity, and reduced agricultural productivity [12]. Furthermore, their spread is strongly influenced by non-climatic factors, such as changes in hydrological regimes, elevated soil salinity, and river regulation [13, 14]. Notably, the invasion of *Tamarix* in North America has been accompanied by frequent interspecific hybridization. Single-locus nuclear DNA markers indicate that approximately 23% of invasive populations in the United States are hybrids of *T. chinensis* and *T. ramosissima*, while genome-wide AFLP (Amplified Fragment Length Polymorphism) analyses reveal hybridization rates as high as 83–87%, with a pronounced latitudinal gradient of introgression [15]. Such hybridization has not been detected in the native range, indicating that these genotypes emerged following introduction [10]. Hybrid-facilitated invasions have been documented in at least 11 plant families, including *Tamarix*, *Populus*, and *Salix*, where hybrids often exhibit enhanced growth, broader environmental tolerance, or accelerated spread [16]. Together, these observations establish hybridization as a characteristic feature of *Tamarix* invasions, providing useful ecological context for interpreting observed patterns of niche overlap and distributional shifts. Given the escalating ecological and management impacts of *Tamarix* invasions across broad geographic regions, and the increasingly important role of climate change in shaping species’ large-scale distribution patterns, we developed a systematic conceptual framework to compare the climatic niches of native and invaded populations and to assess whether niche shifts have occurred during the invasion process. We further aimed to identify the key climatic factors shaping niche differences and to project potential distributional changes of the two species under future climate scenarios. This integrative approach allows us to evaluate niche conservatism versus shifts and to infer possible invasion risks under climate change. This study addresses three key scientific questions: (1) Have the climatic niches of *T. chinensis* and *T. ramosissima* changed during the invasion process? (2) If changes have occurred, how are these changes expressed between native and invaded populations? (3) How will current and future climate change affect the potential geographic distributions of *T. chinensis* and *T. ramosissima*?

## Materials and methods

### Occurrence data

In this study, we focused on *T. chinensis* and *T. ramosissima*, the two taxa that dominate the North American invasion, making them the most ecologically consequential and representative *Tamarix* lineages for analyzing climatic niches and potential distributions. We obtained species distribution records using the dismo package in R and collected data from the Global Biodiversity Information Facility (GBIF, https://www.gbif.org/), with occurrence downloads accessed on 13 June 2024 [17, 18]. After removing duplicate latitude and longitude points, as well as those with unrecognized geographic coordinates, we extracted the distribution data points of *T. chinensis and T. ramosissima* from both Eurasia and North America. Within Eurasia, historical dispersal and human-mediated introductions have resulted in the coexistence of populations with different biogeographic origins, making a fine-scale separation of native and introduced occurrences difficult at the continental level. Accordingly, occurrence records from Eurasia were used to represent the native climatic background of each species, with the aim of characterizing the climatic conditions associated with their region of origin. This continental-scale representation was adopted as a baseline for subsequent comparisons with populations in North America, rather than to resolve detailed dispersal or introduction histories within Eurasia.

**Fig. 1:**
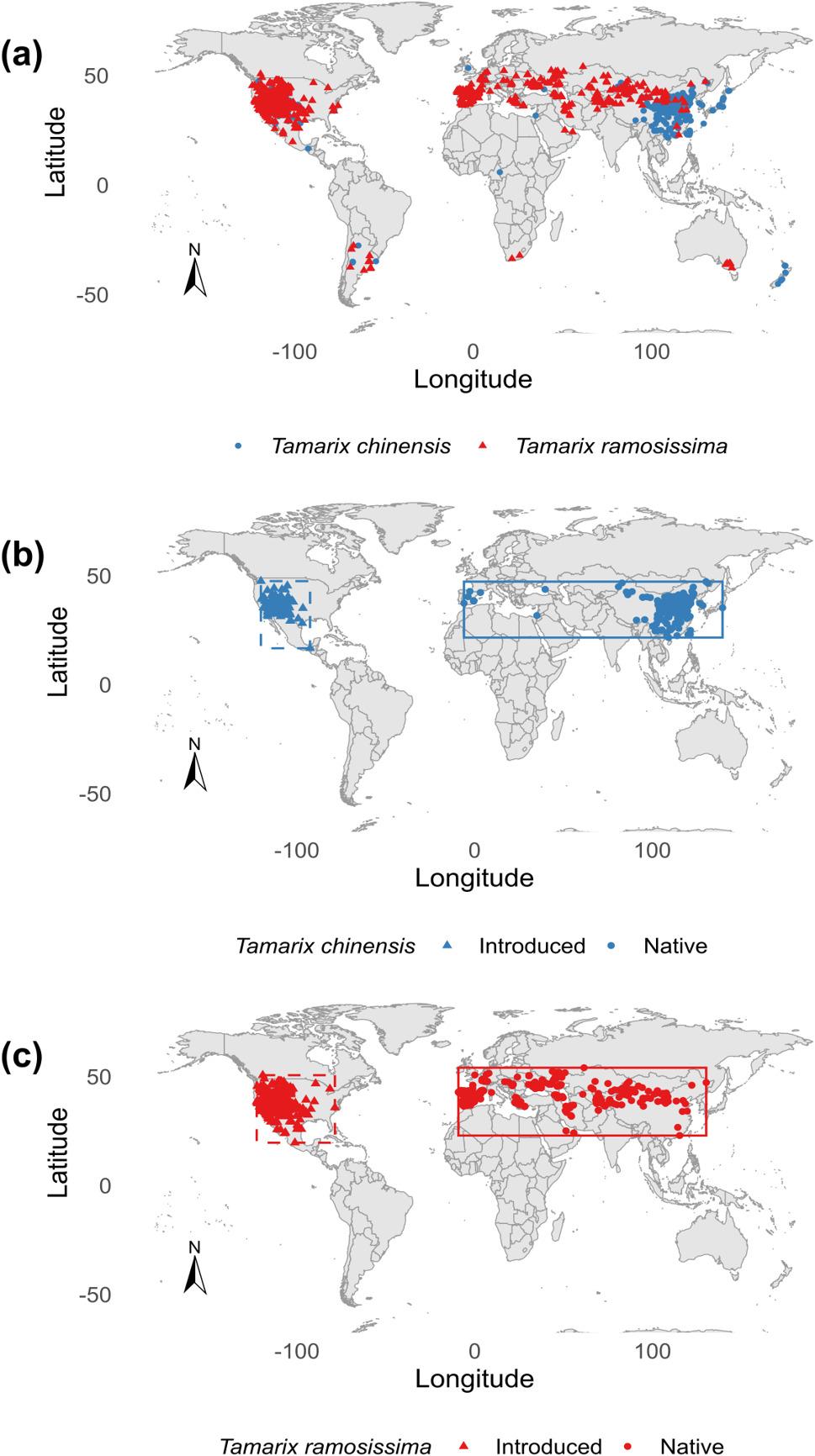
Geographic distribution records of *Tamarix chinensis* and *T. ramosissima*. **a** Global occurrence points of both species; **b** Occurrence points of *T. chinensis*; **c** Occurrence points of *T. ramosissima*. In both panels **b** and **c**, circles represent occurrence records in the native range, while triangles indicate records in the invasive range

To reduce sampling bias in model predictions, occurrence records were randomly thinned to ensure a minimum distance of five kilometers between points, corresponding to the approximate spatial resolution of the climatic layers (5 km at the equator). Using these processed data, 227 and 367 cleaned occurrence points were retained for *T. chinensis* and *T. ramosissima* in Eurasia, respectively, and 231 and 724 points in North America, respectively. Based on these occurrence records, two rectangular calibration areas were delineated to represent regions with the highest density of *Tamarix* records in Eurasia and North America (Fig. 1).

In addition to the single-species datasets, distribution records of *T. chinensis* and *T. ramosissima* were combined to construct an integrated dataset, hereafter referred to as the Species complex. For this Species complex, the environmental background was defined based on the spatial extent of the combined occurrence records, with separate rectangular calibration areas delineated for the native and invaded ranges to maintain consistency with single-species analyses. It should be noted that the species complex serves solely as an analysis unit based on distribution data, intended to complement environmental and spatial analyses of overall ecological niches and potential distributions, providing a comprehensive depiction of climatic suitability patterns across the native and invaded ranges of the two species, and is not intended to identify or distinguish specific hybrid lineages.

### Selection and comparison of climate variables

Because this study focuses on climatic niche dynamics at a transcontinental scale, we restricted predictor variables to bioclimatic factors that are consistently available and ecologically meaningful across broad geographic extents. Although non-climatic factors such as soil salinity, hydrological conditions, and river regulation are known to influence *Tamarix* spread at local and regional scales, these variables are difficult to harmonize across native and invaded ranges in terms of spatial coverage, resolution, and data consistency. Incorporating such variables may also introduce scale mismatches, thereby reducing the transferability and comparability of analytical outcomes among regions. Therefore, this study deliberately focuses on climatic variables to elucidate the role of climate in shaping large-scale niche patterns and potential distributional shifts under climate change. Nineteen bioclimatic variables were selected to investigate the ecological niche differences and suitable habitats of *T. chinensis* and *T. ramosissima* in Eurasia and North America. These variables have a spatial resolution of 2.5 arc-minutes (approximately 5 km²) and were obtained from the WorldClim database (http://www.worldclim.org) [19]. Historical climate data (1970–2000) were used to provide a baseline for model projections. Two Shared Socioeconomic Pathway (SSP) scenarios, SSP1-2.6 and SSP5-8.5, derived from the BCC-CSM2-MR climate model, were used to predict species distributions under future climate conditions. The future period of the 2070s (2061–2080 average) was chosen to assess mid-to-late 21st century climate impacts on *Tamarix* distributions.

Since multicollinearity among predictor variables can lead to overfitting and inaccurate model evaluation, the Pearson correlation coefficient (|r| ≥ 0.85) was used to retain one variable from each highly correlated pair, based on the correlation matrix of the 19 bioclimatic variables (Supplementary Figure S1). After addressing highly correlated variables, eight bioclimatic variables were retained for model construction (Table 1): Annual Mean Temperature (bio1), Mean Diurnal Range (bio2), Temperature Seasonality (bio4), Annual Precipitation (bio12), Precipitation Seasonality (bio15), Precipitation of Driest Quarter (bio17), Precipitation of Warmest Quarter (bio18), and Precipitation of Coldest Quarter (bio19). These variables were chosen based on their ecological relevance for *Tamarix* and their contribution to reducing redundancy in the dataset.

### Statistical analysis of climatic variables

To investigate the ecological niche differences of various populations or species in terms of bioclimatic variables, the eight bioclimatic variables retained after Pearson correlation analysis were used. Values for each variable were extracted from the species distribution points by overlaying the coordinates with global climate raster data, yielding the corresponding climatic factor values for each point.

After data processing, non-parametric statistical methods were applied to evaluate significant differences among groups for each variable, as the data did not necessarily meet normality assumptions. First, the Kruskal-Wallis rank sum test was conducted to assess overall differences of each bioclimatic variable among groups. For variables showing significant differences at a significance level of *p* < 0.05, pairwise comparisons were subsequently performed using Dunn’s test with Bonferroni correction to control for Type I error due to multiple comparisons [20].

### Climatic niche analysis

To investigate the ecological niche dynamics of *Tamarix* species between their native (Eurasia) and invasive (North America) ranges, Principal Component Analysis in environmental space (PCA-env) was employed. This method integrates environmental background data and species occurrence records from the study regions to construct the main climatic gradient space based on the first two principal components. Kernel density estimation was applied to reduce potential sampling bias in the occurrence data.

Niche overlap between the two geographic populations was quantified using Schoener’s *D* metric (ranging from 0 to 1), and permutation tests (α = 0.05) were performed to assess the statistical significance of niche differences [21]. Key climatic variables driving niche shifts were identified by analyzing the variable loadings on the principal component axes, highlighting those contributing significantly to niche divergence.

Occurrence density distributions of native and invasive populations were visualized within the two-dimensional climatic space, and relative shifts of niche centroids were examined. This analysis revealed whether niche shifts occurred and clarified their direction and magnitude, providing evidence for the ecological adaptation and invasion mechanisms of *Tamarix* species.

#### Test of niche equivalence and niche overlap

Niche overlap between geographic ranges was quantified in environmental space using Schoener’s *D* [21] and Warren’s *I* [22] indices, both of which range from 0 (no overlap) to 1 (complete overlap). These metrics provide a quantitative basis for comparing climatic niches across regions.

Based on these overlap metrics, niche equivalency and niche similarity tests were conducted to evaluate the comparability of species’ climatic niches between native and invasive ranges. Both tests rely on a randomization framework but differ in their null hypotheses and permutation procedures [22, 23].

The niche equivalency test examines whether two niches are statistically indistinguishable by testing the null hypothesis that occurrence records from different geographic ranges are drawn from the same underlying climatic niche. Occurrence points from the native and invasive ranges were pooled and randomly reassigned into two groups while preserving the original sample size of each range. This procedure was repeated 1,000 times to generate a null distribution of niche overlap values. The observed overlap was then compared with this null distribution, and values falling outside the 95% confidence interval (*p* < 0.05) led to rejection of the niche equivalency hypothesis, indicating significant niche differences between regions.

In contrast, the niche similarity test evaluates whether the observed niche overlap between two regions is greater or lower than expected by chance given the available environmental background. In this test, the niche of one region was held constant, while niches were randomly sampled from the environmental background of the other region. This process was repeated 1,000 times to generate a distribution of expected overlap values. If the observed overlap was significantly higher than the random expectation (*p* < 0.05), niche similarity was considered greater than expected by chance, supporting climatic niche conservatism. Conversely, non-significant results (*p* > 0.05) suggested that niche similarity did not exceed random expectations, indicating potential niche differentiation.

#### Quantification of niche position and breadth

Principal component axes of environmental variables were extracted using PCA, and niche density grids were constructed within the principal component space. To quantify niche position and breadth, 100 grid cells were randomly sampled from the niche density map, weighted by cell density. Scores of these cells along the first (CP1) and second (CP2) axes were extracted. The median score on each axis represented the niche position, while the variance of the scores represented the niche breadth. This approach allows comparison of niche characteristics across different species or population groups, enabling assessment of potential niche shifts or expansions between native and invasive ranges.

All analyses were conducted in R (version 4.3.3) using the ecospat package [24]. The niche partitioning analysis was based on the combined environmental space of both the native and invasive ranges rather than their intersecting environment.

### Ecological niche model construction and optimization

Ecological niche models (ENMs) were applied to assess the climatic suitability of *Tamarix* species in both native and invasive ranges by linking species occurrence records with environmental variables. These models are based on the assumption of niche conservatism and are widely used in invasion biology to evaluate potential spread and habitat suitability.

MaxEnt (version 3.4.1) was employed to generate distribution models. MaxEnt is a machine learning algorithm based on the maximum entropy principle, relying solely on occurrence data and environmental predictor variables [25]. To reduce variability, each model was run 10 times, and the model best representing the species’ average environmental suitability was retained [26].

#### Model complexity and tuning

Model calibration is essential for improving the predictive accuracy of ecological niche models, as optimizing feature combinations (FCs) and the regularization multiplier (RM) can significantly enhance model performance [27]. The Kuenm R package was employed to fine-tune the MaxEnt model, ensuring optimal performance [28].

Parameter selection involved 31 different feature combinations, encompassing the five basic feature types provided by MaxEnt: linear (L), quadratic (Q), hinge (H), product (P), and threshold (T), resulting in 1,240 unique parameter configurations. The regularization multiplier (RM) was set within a range of 0.1 to 4, with an increment of 0.1, yielding 40 different RM values to comprehensively explore model complexity. All 1,240 models were trained using the distribution data of the two species, and model evaluation was conducted using the corrected Akaike Information Criterion (ΔAICc < 2) to select models with the least information loss [29].

This extensive testing of FC and RM combinations allowed selection of the best-performing model for predicting the potential distribution of *T. chinensis* and *T. ramosissima*, ensuring high predictive accuracy while avoiding overfitting.

#### Reciprocal distribution modeling

The ecological niche modeling (ENM) approach [30] was applied to assess the potential distribution of *T. chinensis* and *T. ramosissima* in both their native and invasive ranges. This approach involves two steps. First, a model is trained using distribution data from the native range (e.g., Eurasian range of *Tamarix*) and projected onto the invasive range (e.g., North American range of *Tamarix*) to predict suitability in the new environment. Second, a model is trained using distribution data from the invasive range and projected back onto the native range to evaluate adaptability to the original environment. This reciprocal modeling framework facilitates understanding of niche conservatism and species’ responses to environmental changes.

#### Modeling under current and future climate scenarios

To evaluate the effects of climate change on species distributions, ecological niche models were developed for *T. chinensis*, *T. ramosissima*, and the species complex using the eight bioclimatic variables retained after Pearson correlation analysis. For each taxon, models were constructed for current climatic conditions and for future projections for the 2070s (2061–2080 average) under SSP1-2.6 and SSP5-8.5 scenarios based on the BCC-CSM2-MR climate model. The resulting MaxEnt outputs were binarized using the Maximum Training Sensitivity plus Specificity threshold (MTP) to delineate suitable and unsuitable habitats. Based on this classification, suitable habitat areas were measured using the entire continents of Eurasia and North America as boundaries, and changes under different climate scenarios were compared to assess the effects of climate change on potential species distributions.

#### Model evaluation

The predictive performance of the Ecological Niche Models was evaluated using the area under the receiver operating characteristic (ROC) curve (AUC) [31]. The ROC curve illustrates how the proportion of correctly predicted presences (true positives) changes relative to incorrectly predicted presences (false positives) under different decision thresholds. The AUC summarizes this information into a single, threshold-independent value, representing the probability that the model assigns a higher suitability score to a true presence than to a pseudo-absence, and is therefore widely used to evaluate model discrimination performance [32].

According to the standardized evaluation system, an AUC value > 0.9 indicates excellent predictive performance, while a value above 0.8 represents high predictive accuracy. Conversely, the closer the AUC value is to 0.5, the poorer the model’s predictive ability [33].

## Results

### Direct comparison of environmental variables

**Fig. 2:**
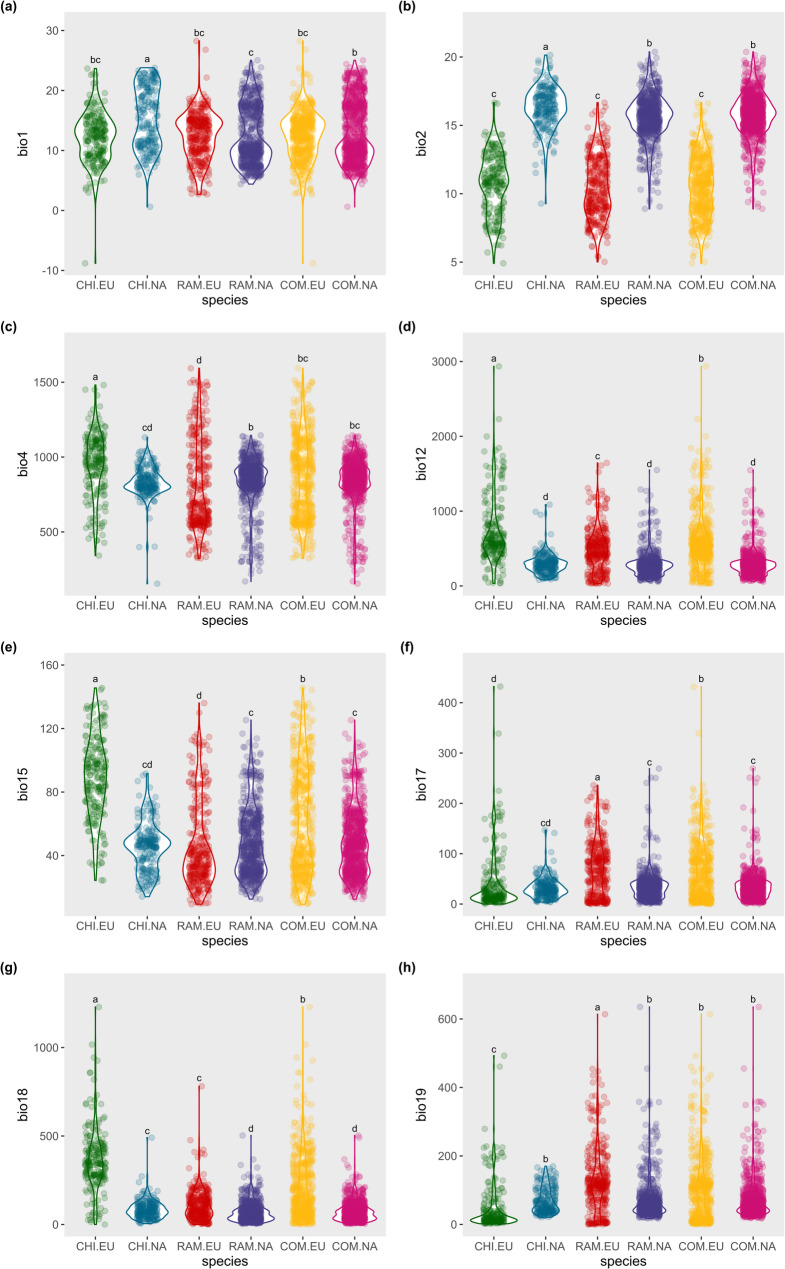
Comparison of occurrence-associated bioclimatic variables of *Tamarix chinensis*, *T. ramosissima*, and their combination species in the introduced and native ranges. Violin plots show the distributions of selected bioclimatic variables (bio1, bio2, bio4, bio12, bio15, bio17, bio18, and bio19) associated with species occurrences across different species and regions. Different letters above the plots indicate statistically significant differences among groups based on Kruskal–Wallis tests followed by post hoc pairwise comparisons (*α* = 0.05). The horizontal axis represents different *Tamarix* categories within various regions: CHI.EU indicates *T. chinensis* in the Eurasian continent, CHI.NA indicates *T. chinensis* in North America, RAM.EU indicates *T. ramosissima* in the Eurasian continent, RAM.NA indicates *T. ramosissima* in North America, COM.EU represents the combination of both species in the Eurasian continent, and COM.NA represents the combination of both species in North America

The significance of differences in occurrence-associated bioclimatic variables between the native (Eurasia) and invasive (North America) ranges for *T. chinensis*, *T. ramosissima*, and the Species complex are summarized in Table 1, while the distributions of these variables are illustrated in Fig. 2.

For *T. chinensis*, significant differences were observed for all bioclimatic variables except bio17 (precipitation of the driest quarter). Invasive populations experienced higher annual mean temperature (bio1) and diurnal temperature range (bio2), while temperature seasonality (bio4) and most precipitation-related variables (bio12, bio15, bio18) were lower compared to native populations. Precipitation of the coldest quarter (bio19) was slightly lower in native populations.

For *T. ramosissima*, all variables differed significantly except bio1 (annual mean temperature). Invasive populations showed higher diurnal temperature range (bio2), whereas temperature seasonality (bio4) was lower. Most precipitation variables (bio12, bio17, bio18, bio19) were lower in invasive populations, except for precipitation seasonality (bio15), which was higher in invasive populations.

For the Species complex, significant differences were observed for all variables except bio1, bio4, and bio19. Invasive populations were characterized by higher diurnal temperature range (bio2) and lower precipitation-related variables (bio12, bio15, bio17, bio18) compared to native populations.

Overall, these patterns indicate consistent shifts in temperature and precipitation variables between native and invasive ranges, highlighting key climatic factors that may contribute to the successful establishment of *Tamarix* species in North America.

**Table 1:** Occurrence-associated bioclimatic variables of *Tamarix* species in native and invasive ranges

Bioclimatic Variable	T. chinensis (EU vs. NA)	T. ramosissima (EU vs. NA)	Species complex (EU vs. NA)
bio1 (Annual Mean Temperature)	*	ns	ns
bio2 (Mean Diurnal Range)	*	*	*
bio4 (Temperature Seasonality)	*	*	ns
bio12 (Annual Precipitation)	*	*	*
bio15 (Precipitation Seasonality)	*	*	*
bio17 (Precipitation of Driest Quarter)	ns	*	*
bio18 (Precipitation of Warmest Quarter)	*	*	*
bio19 (Precipitation of Coldest Quarter)	*	*	ns

Note: “*” indicates a statistically significant difference between EU and NA (α = 0.05), whereas “ns” denotes no significant difference. EU = Eurasia; NA = North America

### Niche comparisons in environmental space

**Fig. 3:**
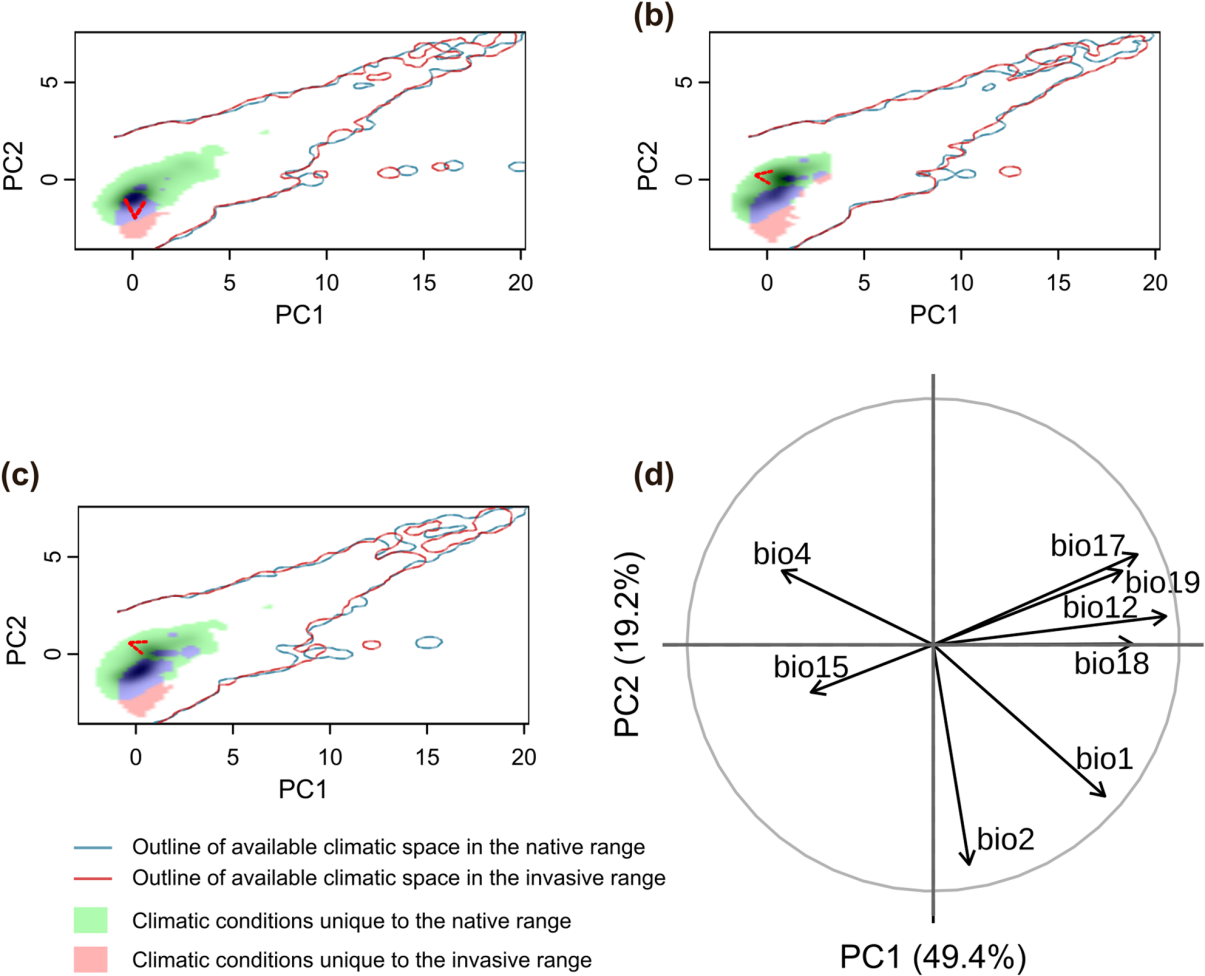
Principal Component Analysis (PCA) of climatic niche variation in *Tamarix* species. **a** *T. chinensis*; **b** *T. ramosissima*; **c** Species complex; **d** Correlation circle showing the contribution of each variable to the niche space defined by the first two principal component axes. Blue and red contour lines delineate the density-based outlines of the available climatic space in the native (Eurasia) and invasive (North America) ranges, respectively. The red arrow indicates the shift in the centroid of the available climatic space between ranges. Green shaded cells indicate climatic conditions available in the native range but not represented by occurrences in the invasive range. Pink shaded cells indicate climatic conditions represented by occurrences in the invasive range but not available in the native range. All elements are displayed in a reduced PCA climatic space and represent different aspects of climatic space structure rather than strict spatial containment

Principal component analysis (PCA) revealed that the first two components accounted for 68.6% of the total climatic variation, with PC1 and PC2 explaining 49.4% and 19.2%, respectively (Fig. 3d). PC1 primarily represented a precipitation gradient, and PC2 represented temperature variation. Since PCA axes are oriented arbitrarily, the direction of climatic gradients was interpreted based on the loading patterns rather than the sign of the axes. The loadings indicated that positive PC1 corresponds to wetter conditions, whereas positive PC2 indicates cooler environments. The climatic spaces occupied by *T. chinensis*, *T. ramosissima*, and the Species complex in the native (Eurasia) and invasive (North America) ranges are shown in Fig. 3a to 3c. The corresponding niche metrics are summarized in Tables 2 and 3.

**Table 2:** Ecological niche overlap (Schoener’s *D* and Hellinger’s *I*) among *Tamarix chinensis*, *T. ramosissima*, and the Species complex across native and invasive ranges

Species 1	Species 2	Niche Overlap
*T. chinensis* (native)	*T. chinensis* (invasive)	*D* = 0.11, *I* = 0.23
*T. ramosissima* (native)	*T. ramosissima* (invasive)	*D* = 0.13, *I* = 0.28
Species complex (native)	Species complex (invasive)	*D* = 0.15, *I* = 0.31
*T. chinensis* (native)	*T. ramosissima* (native)	*D* = 0.64, *I* = 0.84
*T. chinensis* (invasive)	*T. ramosissima* (invasive)	*D* = 0.63, *I* = 0.87

Note: *D* represents Schoener’s *D*, and *I* refers to Hellinger’s-based *I* statistic of ecological niche overlap

**Table 3:** Ecological niche position and breadth of *Tamarix chinensis, T. ramosissima*, and the Species complex in Eurasia and North America based on the first two principal components

Species	Position 1	Breadth 1	Position 2	Breadth 2
*T. chinensis* (native)	-0.79	1.65	4.76	0.59
*T. chinensis* (invasive)	-0.32	0.22	6.01	0.32
*T. ramosissima *(native)	-0.55	0.98	4.36	0.55
*T. ramosissima* (invasive)	-0.32	0.52	5.84	0.56
Species complex (native)	-0.55	1.23	4.53	0.59
Species complex (invasive)	-0.32	0.38	5.84	0.38

Note: “Position1” and “Position2” indicate the species’ mean niche positions along the first and second principal component axes, respectively. “Breadth1” and “Breadth2” refer to niche breadth along PC1 and PC2

The ecological niche overlap between the native and invasive ranges was low for both individual species, suggesting that they occupy substantially different climatic niches across regions. In contrast, analysis of the Species complex showed increased overlap, indicating greater climatic consistency in the merged distribution range (Table 2).

Niche centroid shift analysis showed that all three groups experienced a significant leftward shift along the first principal component axis (PC1), indicating that the invasive range is characterized by drier climatic conditions compared to the native range. Additionally, *T. chinensis* exhibited a pronounced downward shift along the second principal component axis (PC2), suggesting a tendency toward drier and warmer environments in the invasive range. These centroid shifts reflect changes in environmental preferences between the native and invasive ranges and support the hypothesis of ecological niche reconstruction during invasion.

Niche breadth analysis revealed a clear contraction along PC1 across all groups, most notably in *T. chinensis*. While most groups also showed reduced niche breadth along PC2, *T. ramosissima* remained relatively stable, suggesting consistent climatic tolerance in the invasive range (Table 3).

### Reciprocal distribution modeling

**Fig. 4:**
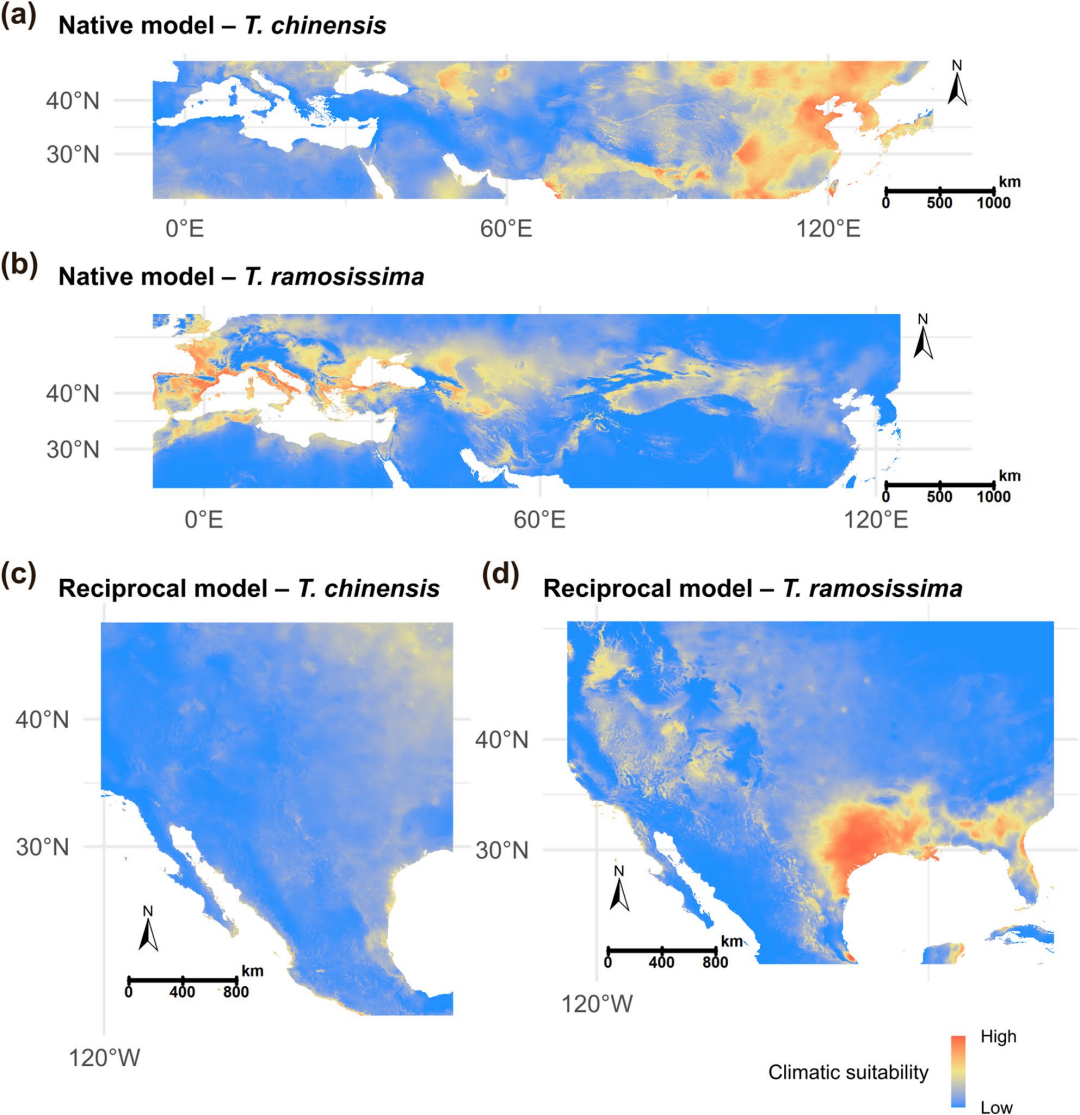
Distribution models of *Tamarix chinensis* and *T. ramosissima* calibrated in the native range (**a**, **b**) and projected to the invasive range (**c**, **d**). **a** *T. chinensis* distribution model calibrated in the native range. **b** *T. ramosissima* distribution model calibrated in the native range. **c** Reciprocal distribution model for *T. chinensis* trained in the invasive range. **d** Reciprocal distribution model for *T. ramosissima* trained in the invasive range

The native Maxent model indicates that *T. chinensis* is primarily distributed in eastern China, northern China, and Central Asia, with particularly high suitability in the North China Plain, the lower reaches of the Yellow River, and the eastern coastal regions of China. *T. ramosissima*, on the other hand, shows high suitability in southern Europe, Central Asia, and northern and northwestern China, with dense populations especially along the Mediterranean coast and in North China (Fig. 4). Both *Tamarix* species are generally associated with arid, semi-arid, and temperate climates. Models calibrated with invasive-range occurrence data show that both species exhibit high suitability in the southwestern United States and northwestern Mexico (Appendix Fig. 2). These regions are characterized by arid and Mediterranean-type climates, with sparse and uneven rainfall and large temperature fluctuations.

However, when the models were projected across regions to construct reciprocal niche models, substantial discrepancies emerged between the predictions and the actual species distributions. The native-range models failed to accurately predict the species’ distributions in the invasive range, and the invasive-range models similarly failed to capture their distributions in the native range.

### Predicting future potential distribution

**Fig. 5:**
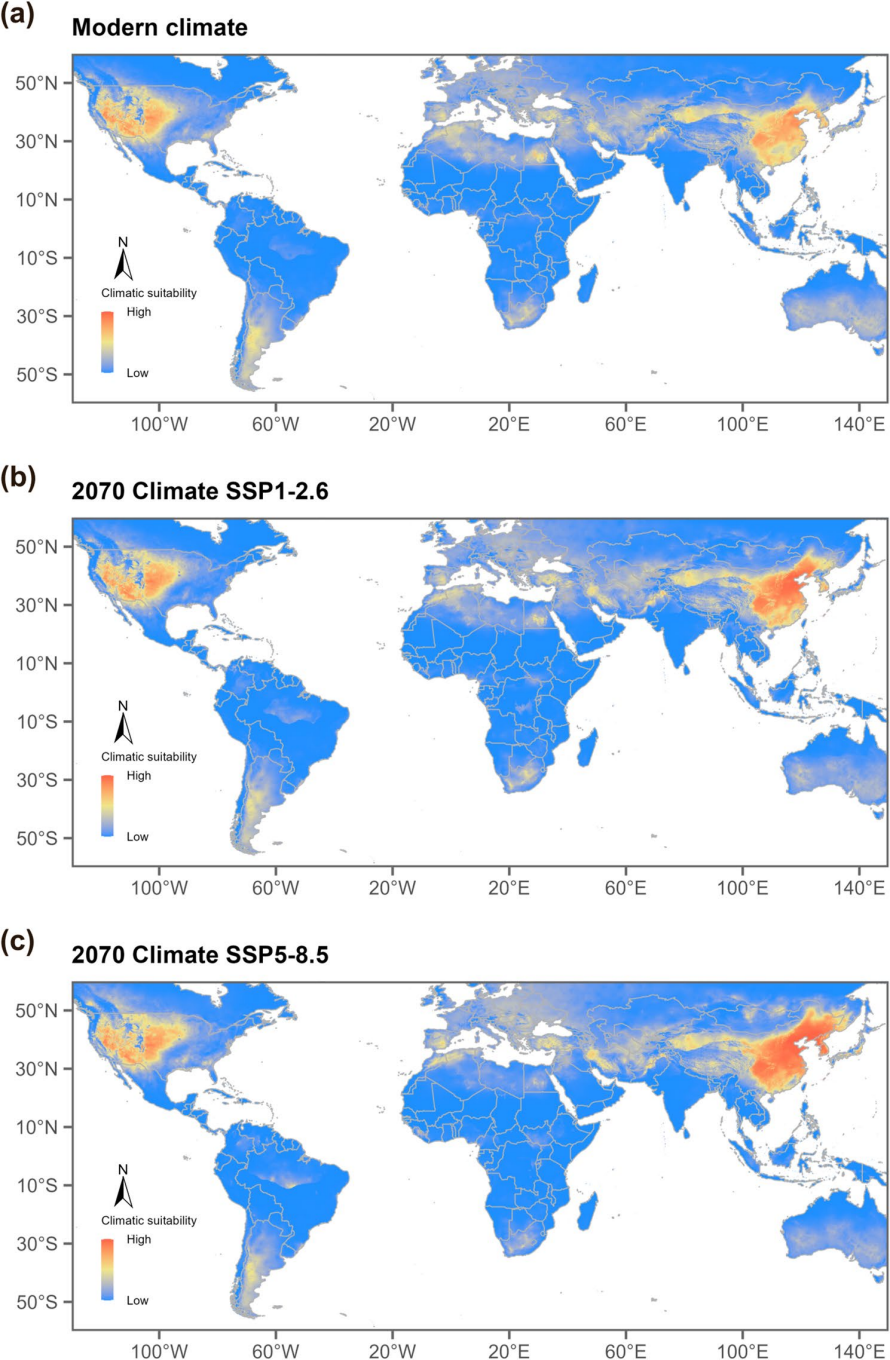
Modeled global habitat suitability for *Tamarix chinensis* under **a** present-day climate, **b** future climate in 2070 under the SSP1-2.6 scenario, and **c** future climate in 2070 under the SSP5-8.5 scenario. Red indicates the highest predicted habitat suitability. Map lines depict the study area and do not necessarily represent officially recognized national boundaries

**Fig. 6:**
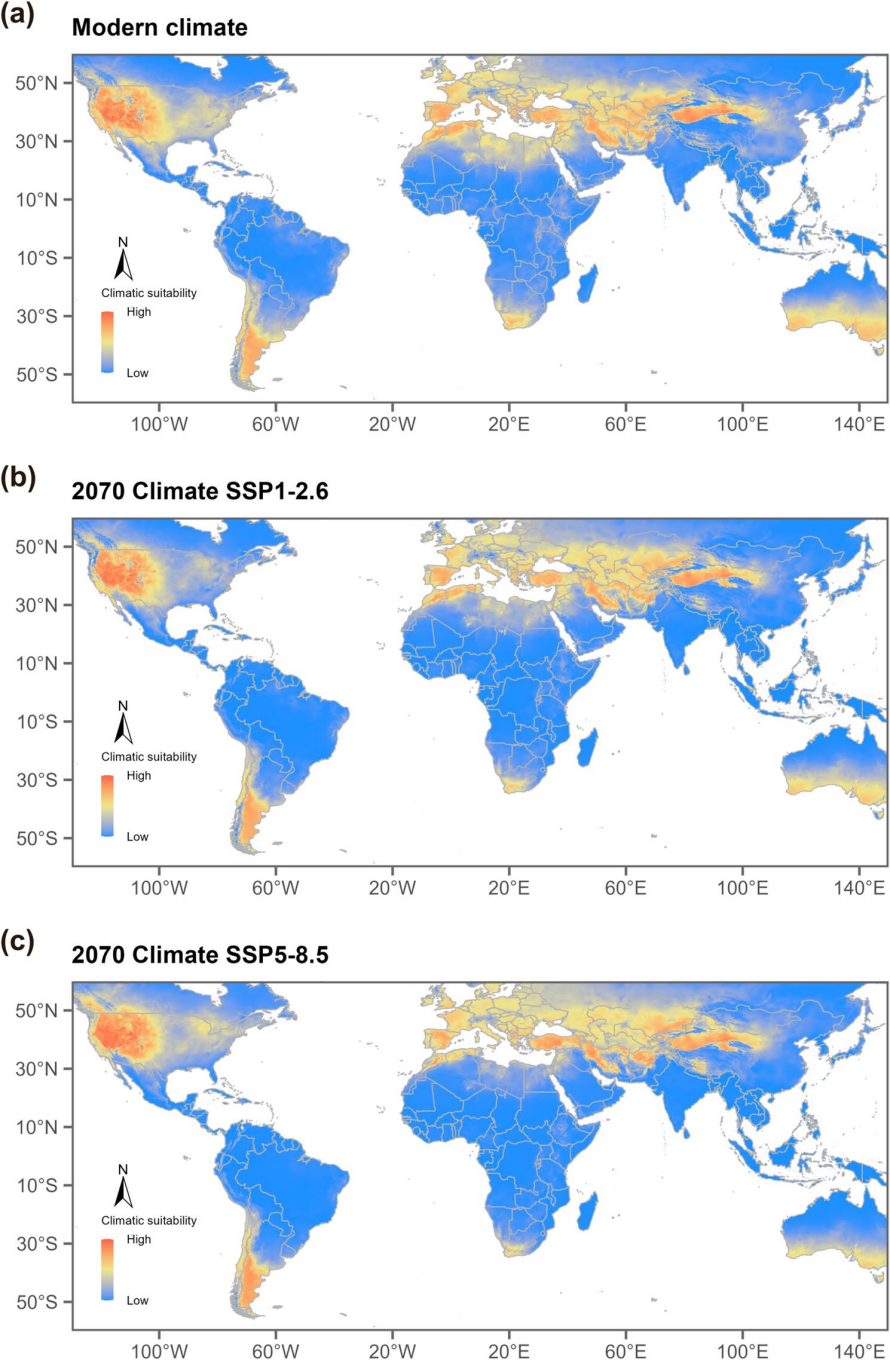
Modeled global habitat suitability for *Tamarix ramosissima* under **a **present-day climate, **b** future climate in 2070 under the SSP1-2.6 scenario, and **c** future climate in 2070 under the SSP5-8.5 scenario. Red indicates the highest predicted habitat suitability. Map lines depict the study area and do not necessarily represent officially recognized national boundaries

The ecological niche models developed in this study indicate that the current potential distributions of *T. chinensis* (Fig. 5) and *T. ramosissima* (Fig. 6) closely match known distribution data, confirming the reliability of the models. To assess potential species responses under contrasting future climate conditions, two Shared Socioeconomic Pathway (SSP) scenarios were considered, with SSP1-2.6 representing a low-emission, sustainable development pathway and SSP5-8.5 representing a high-emission, fossil fuel-driven pathway. Under these future climate scenarios for the 2070s (2061–2080 average), suitable habitats exhibit clear latitudinal differences. Regions north of 30°N are projected to experience a northward shift of suitable habitats, while areas south of 30°N generally show a contraction. In traditionally suitable regions, such as the North China Plain in East Asia and the southwestern United States, suitable habitats are projected to move northward under both SSP1-2.6 and SSP5-8.5. In particular under SSP5-8.5, the northward movement of suitable-area boundaries in eastern Eurasia and western North America is expected to be more pronounced. (Figs. 5c and 6c).

Based on our estimates of potential suitable habitat area, in the native range (Eurasia), by 2070, *T. chinensis* is nearly stable under SSP1-2.6, *T. ramosissima* decreases slightly, and the species complex increases slightly. Under SSP5-8.5, *T. chinensis* and the species complex further increase, while *T. ramosissima* continues to decline. In the invasive range (North America), under SSP1-2.6, *T. chinensis* decreases slightly, *T. ramosissima* remains nearly stable, and the species complex increases; under SSP5-8.5, all three taxa show pronounced expansion. (Supplementary Table S1).

## Discussion

### *Tamarix* populations in the invasive range exhibit stronger adaptation to arid climates

This study focused on the core distribution areas of *Tamarix chinensis* and *Tamarix ramosissima* in Eurasia and North America. Although populations of these species within Eurasia include both native and historically introduced lineages, at a macro-geographical scale, the spatial separation, climatic background, and long-term ecological conditions between Eurasia and North America far exceed the local climatic heterogeneity within Eurasia. This intercontinental scale difference allows Eurasian occurrence records to represent the primary climatic background of the species, providing a robust macro-scale reference for comparisons with populations in the North American invasive range [34].

Both study regions are located between 30° and 50° N latitude and exhibit comparable interannual temperature fluctuations and seasonal precipitation patterns, indicating broadly similar climatic contexts. Given the strong physiological and demographic sensitivity of *Tamarix* to temperature, such similarity in thermal regimes between the native and invaded ranges likely constitutes an important climatic precondition facilitating its successful invasion in North America. Indeed, when historical climate data are considered, extreme temperature conditions—particularly winter minima and summer maxima—remain critical thresholds constraining the realized distribution of these species [35, 36]. Reproductive success in *Tamarix* is highly temperature-dependent, and deviations from optimal thermal ranges can impair floral development, fertilization, and seed maturation, as well as disrupt enzymatic activity and photosynthetic efficiency, ultimately limiting population persistence [37–39].

Despite this overall similarity in temperature regimes, comparisons between native and invasive ranges reveal pronounced shifts in climatic niches, especially along temperature-related dimensions. For both *T. chinensis* and *T. ramosissima*, invasive populations are associated with higher diurnal temperature range (Bio2) and lower temperature seasonality (Bio4), and invasive populations of *T. chinensis* additionally experience higher annual mean temperature (Bio1). These shifts may extend the growing season, reduce winter mortality, and enhance competitive ability under arid conditions [40, 41]. A greater diurnal temperature range may also promote nocturnal dew formation, thereby reducing transpiration demand and limiting salt accumulation [42], while specialized salt glands further alleviate salinity stress [43]. Reduced temperature seasonality may further induce phenological adjustments, such as earlier budburst or delayed senescence, indirectly favoring invasion by increasing competitive advantage relative to native species.

Precipitation patterns further differentiate the invasive habitats of the two *Tamarix* species. Overall, most precipitation-related variables (Bio12 and Bio18, and Bio17 in *T. ramosissima*) are lower in the invasive range than in the native range, indicating generally drier conditions. However, precipitation seasonality (Bio15) and precipitation of the coldest quarter (Bio19) exhibit contrasting changes. For *T. chinensis*, Bio15 decreases while Bio19 increases, whereas *T. ramosissima* shows the opposite trend. These differences may reflect distinct strategies for coping with local water availability. Specifically, *T. chinensis* has flexible phenology, allowing it to adjust growth cycles according to rainfall timing, and a well-developed deep root system that could efficiently utilize intermittent rainfall and episodic flood events [44]. In contrast, the increased precipitation seasonality in the invasive range of *T. ramosissima* may reflect adaptation to concentrated rainfall events, and during this process the species maintains water balance and metabolic function through stomatal regulation and osmotic adjustment [45, 46].

These shifts in niches related to temperature and precipitation suggest that *Tamarix* populations in North America are responding to novel environmental conditions through adaptive flexibility, which may enhance their establishment and persistence in arid and semi-arid regions.

### Niche expansion occurred during the invasion of *Tamarix* species

The ecological niche defines the range of environmental conditions under which a species can survive, grow, and reproduce. It encompasses factors such as temperature, humidity, water availability, and resource distribution, as well as the species’ ability to adapt to novel environments [47]. Regional climatic variation can promote intraspecific differentiation by selecting for phenotypes suited to new environmental contexts, potentially resulting in expansion, contraction, or reconfiguration of ecological niches during biological invasions [48].

In this study, PCA-env analyses revealed substantial differences in niche space between native and invasive populations of *T. chinensis* and *T. ramosissima*. Low niche overlap between ranges indicates that both species have adjusted their ecological niches to the environmental conditions of North America. Niche equivalency and similarity tests confirmed significant differences between continents, providing no support for strict niche conservatism.

Reciprocal distribution models further corroborate these results. Models trained on native-range occurrences poorly predicted invasive distributions, and vice versa, with invasive-range models erroneously projecting suitable habitats in uncolonized areas such as Gulf of Mexico coastal wetlands. These mismatches likely reflect niche shifts rather than simple model error, influenced by the species’ affinity for riverbanks and wetlands.

The observed niche shifts are likely driven by a combination of high dispersal capacity, pre-adaptation to arid and saline environments, and ecological release from natural enemies in the invaded range. Similar strategies are seen in other halophytic invaders; for example, *Mesembryanthemum crystallinum* modifies local soil salinity through root-mediated salt secretion, facilitating colonization of arid regions in the western United States [49]. Genomic evidence, including polyploidy, gene flow among populations, and chromosomal variations, further supports enhanced environmental adaptability in *Tamarix* [50].

Interestingly, while niche shifts occurred, niche breadth analyses indicate a general contraction in the climatic niches of both species during invasion, suggesting increased specialization to certain environmental conditions in North America. Analysis of niche centroid positions provides additional support for directional shifts. All three taxa showed a leftward displacement along the first PCA axis (associated with precipitation), reflecting adaptation to drier environments. *T. chinensis* also exhibited a downward shift along the second axis, corresponding to warmer conditions. Together, these changes in centroid positions and niche breadth capture both the direction and extent of ecological niche restructuring during invasion.

### Climate change may locally increase the invasion potential of *Tamarix*

The impact of climate change on the distribution of invasive species has become an important research topic. Accurately predicting potential distributions is crucial for identifying high-risk areas and guiding effective management strategies [51]. Ecological niche models (ENMs), particularly MaxEnt, are effective for capturing complex, nonlinear relationships between species and environmental variables, making them powerful tools for predicting invasion risks under climate change [52, 53].

This study is the first to apply MaxEnt to simulate both current and future global distributions of *T. chinensis* and *T. ramosissima*. Models exhibited high predictive performance (AUC > 0.8), and predicted climatic niches closely matched observed occurrences, providing a reliable basis for projecting future range shifts [25].

Future climate change may lead to a northward shift in the suitable habitats of *T. chinensis* and *T. ramosissima*, particularly within traditionally suitable regions such as eastern Eurasia and the southwestern United States. At the same time, areas that currently exhibit low climatic suitability, such as northeastern China and central United States, may become emerging habitats for *Tamarix*. This pattern reflects the general tendency of invasive species to migrate toward higher latitudes and elevations in response to rising temperatures [54]. Notably, our quantitative analysis of suitable habitat areas in both native and invasive ranges indicates that by 2070, the native ranges of *Tamarix chinensis*, *T. ramosissima*, and the Species complex show species-specific changes, whereas suitable habitat areas in the invasive ranges generally expand. Considering the overall northward shift of these three taxa, we infer that suitable habitats may undergo spatial reorganization, with local patterns experiencing dynamic adjustments rather than uniform expansion. Accordingly, management strategies should simultaneously address emerging habitats and existing invaded areas, emphasizing early-stage surveillance and rapid response, particularly through focused monitoring of riparian corridors and other disturbance-prone habitats where *Tamarix* establishment is most likely. Preventive measures aimed at limiting initial establishment and reducing the likelihood of secondary spread are especially important, as interventions are generally more effective and less costly before *Tamarix* populations become widely established [55, 56].

According to the IPCC Sixth Assessment Report [57], the frequency and intensity of extreme high-temperature events in the Northern Hemisphere are projected to increase, often accompanied by more frequent droughts. These climatic changes are expected to relax thermal constraints at higher latitudes, thereby increasing the climatic suitability of these regions for *Tamarix* establishment. This response is consistent with the strong physiological adaptability of *Tamarix*, including deep root systems that access groundwater and dynamic stomatal regulation that maintains water balance, allowing persistence and spread under hot and dry conditions [13]. However, the extent to which warming promotes invasion is unlikely to be uniform across regions, as precipitation regimes may substantially modulate these effects. While rising temperatures may facilitate northward range expansion, areas characterized by higher precipitation may impose constraints on establishment and growth [58]. Consequently, future distributions of *T. chinensis* and *T. ramosissima* are likely to reflect the combined influence of temperature increases and spatial heterogeneity in precipitation. Overall, continued climate warming is expected to elevate invasion potential in high-latitude regions, underscoring the importance of integrating climate-based niche projections with forward-looking monitoring and management strategies to better anticipate and mitigate future *Tamarix* invasions.

Climate warming is likely to increase the invasion potential of *T. chinensis* and *T. ramosissima* in high-latitude regions, driven by their physiological adaptability and ongoing shifts in climatic suitability. Together, these findings underscore the value of integrating climate-based niche projections with forward-looking monitoring and management efforts to better anticipate and mitigate future *Tamarix* invasions under continued climate change.

### Climatic response patterns and management strategy assessment based on integrated data

Ecological niche analyses based on the integrated distribution data of *T. chinensis* and *T. ramosissima* revealed significant differences in most bioclimatic variables between native and invasive ranges. Precipitation-related variables (bio12, bio15, bio17, bio18) were consistently lower in the invasive range, while the mean diurnal temperature range (bio2) was higher, suggesting adaptive adjustments to drier and more thermally variable conditions. Certain variables (e.g., bio1, bio4, bio19) that were significant in analyses of individual species were no longer significant when data from both species were combined, indicating that integration may mask species-specific responses and reflect a more generalized pattern of niche adaptation.

Niche overlap analyses further showed that *T. chinensis* and *T. ramosissima* maintain highly overlapping ecological spaces across regions, pointing to convergent strategies and similar environmental tolerances. The Species complex model exhibited slightly higher overlap between native and invasive ranges than single-species models, suggesting that the merged taxon possesses broader and more stable climatic tolerance. Although the niche breadths along the two principal component axes slightly narrowed after integration, the changes were minimal, and the centroid shifts remained in the same direction, indicating consistent directional niche shifts.

These patterns indicate that, in regions where the two species coexist or frequently hybridize, species-level distinctions may not fully reflect ecological reality, thereby increasing the complexity of long-term management. Hybridization can blur the morphological and ecological boundaries of the parental species, making accurate identification and species-specific management difficult in practice [10]. Hybridization studies [59] have shown that hybrids may occupy ecological niches distinct from either parent, exhibiting altered climatic tolerances and habitat preferences. Such niche restructuring can lead to heterogeneous responses to environmental conditions and management interventions, thereby increasing uncertainty in spread dynamics predictions and reducing the effectiveness of strategies targeting parental species individually. Therefore, in cases where species boundaries are blurred or hybrid taxa are present, our analyses based on integrated distribution data of the two species propose a hypothetical discussion that, in hybrid or coexisting regions, the two species could be considered as a unified ecological management unit.

Moreover, integrating distribution data can enhance model reliability, as larger sample sizes improve the predictive accuracy of species distribution models [60]. The combined models generate more continuous and robust predictions of suitable habitats, better capturing the overlapping climatic tolerances and coexistence patterns of the two species. In native ranges, the Species complex model predicts an increase in suitable habitat under future climate scenarios, while the individual species exhibit species-specific responses, including minor fluctuations or declines, likely reflecting the stabilizing effect of integrating data from both species. These integrated approaches provide a robust technical framework in both theoretical and operational terms, allowing exploration of potential shifts in habitat suitability patterns under strong niche overlap. It should be noted, however, that this approach is not intended to infer specific hybridization events and does not represent discrete hybrid taxa, as hybridization is a genetic process that cannot be resolved solely from distribution data.

Finally, it should be noted that, although *T. chinensis* and *T. ramosissima* exhibit a high degree of overlap in both their ecological niches and geographic distributions across native and invasive ranges, significant differences remain, indicating that even under conditions of high ecological similarity, they may retain ecologically meaningful distinctions. Therefore, the hypothesis of treating them as a unified ecological management unit should be applied cautiously, taking into account taxonomic accuracy and regional ecological differences. Future research could further integrate gene flow analyses and ecological interaction data to verify whether they constitute a stable evolutionary unit.

## Conclusion

This study indicates that *T. chinensis* and *T. ramosissima* have experienced notable niche contraction during their invasion from Eurasia to North America, accompanied by shifts in temperature- and precipitation-related distributions. These changes suggest that the species may have adjusted their ecological strategies to cope with novel environmental conditions, enhancing their potential to establish and spread in arid and semi-arid regions. MaxEnt simulations under current and future climate scenarios (SSP1-2.6 and SSP5-8.5 for 2070) show that suitable habitats of *Tamarix* species in the Northern Hemisphere shift toward higher-latitude regions. The rapid environmental adaptability of these two species under climate change may enhance their invasiveness, highlighting the urgency of implementing adaptive management strategies to mitigate ecological impacts. These results provide a quantitative basis for assessing the future invasion potential of *Tamarix* species and inform the development of targeted management strategies under changing climatic conditions.

## Supplementary Information

Supplementary Material 1. Supplementary Figure S1: Heatmap of Pearson correlation coefficients among 19 bioclimatic variables used in this study. Colors from blue to red indicate negative and positive correlations, with intensity reflecting correlation strength (−1 ≤ r ≤ 1). Highly correlated variables were identified to guide variable selection for ecological niche modeling and reduce multicollinearity. Supplementary Figure S2: Distribution models of Tamarix chinensis and T. ramosissima calibrated in the invasive range (a, b) and projected to the native range (c, d). Panel (a) shows the distribution model of T. chinensis calibrated in the invasive range, while panel (b) shows the model for T. ramosissima calibrated in the invasive range. Panels (c) and (d) display reciprocal distribution models trained in the native range for T. chinensis and T. ramosissima, respectively. Supplementary Figure S3: Modeled global habitat suitability for the species complex under three climate scenarios: (a) present-day climate, (b) future climate in 2070 under SSP1-2.6, and (c) future climate in 2070 under SSP5-8.5. Areas shown in red indicate the highest predicted habitat suitability. Map boundaries indicate the study area and do not necessarily correspond to officially recognized national borders. Supplementary Table S1: Suitable habitat areas (km²) for Tamarix species and the Species complex under current and future climate scenarios (SSP1-2.6 and SSP5-8.5 for 2070) in Eurasia and North America.

## Data Availability

Species occurrence data supporting the findings of this study are openly available in the Global Biodiversity Information Facility (GBIF) on 13 June 2024. Occurrence records for *T. chinensis* (DOI: 10.15468/dl.tbxbtd) and *T. ramosissima* (DOI: 10.15468/dl.rntnkt) were downloaded from GBIF. Environmental variables were obtained from WorldClim (https://www.worldclim.org/), accessed on 13 June 2024. which provides publicly accessible climate data. All datasets used are openly available and sufficient to reproduce the study.

## References

[1] Deslippe JR, Veenendaal JA. Plant Invasions in a Changing Climate: Reshaping Communities, Ecosystem Functions, and Services. Annu Rev Ecol Evol Syst. 2025;56(1):571–96.

[2] Vilà M, Espinar JL, Hejda M, Hulme PE, Jarošík V, Maron JL, et al. Ecological impacts of invasive alien plants: a meta-analysis of their effects on species, communities and ecosystems: Ecological impacts of invasive alien plants. Ecol Lett. 2011 July;14(7):702–8. 10.1111/j.1461-0248.2011.01628.x 21592274

[3] Pyšek P, Richardson DM. Invasive Species, Environmental Change and Management, and Health. Annu Rev Environ Resour. 2010;35(1):25–55.

[4] Booth TH, Nix HA, Busby JR, Hutchinson MF. bioclim: the first species distribution modelling package, its early applications and relevance to most current MaxEnt studies. Divers Distrib. 2014;20(1):1–9.

[5] Peterson AT. Ecological niche conservatism: a time-structured review of evidence. J Biogeogr. 2011;38(5):817–27.

[6] Yuan Y, Tang X, Liu M, Liu X, Tao J. Species Distribution Models of the Spartina alterniflora Loisel in Its Origin and Invasive Country Reveal an Ecological Niche Shift. Front Plant Sci. 2021;12:738769. 34712259 10.3389/fpls.2021.738769 PMC8546191

[7] Nekrasova O, Pupins M, Tytar V, Fedorenko L, Potrokhov O, Škute A, et al. Assessing Prospects of Integrating Asian Carp Polyculture in Europe: A Nature-Based Solution under Climate Change? Fishes. 2024;9(4):148.

[8] Araya T, Mlahlwa AV, Elbasit MAMA, Newete SW. The impact of Tamarix invasion on the soil physicochemical properties. Sci Rep. 2022;12(1):5750. 35388109 10.1038/s41598-022-09797-3 PMC8986823

[9] Di Tomaso JM. Impact, Biology, and Ecology of Saltcedar (*Tamarix* spp.) in the Southwestern United States. Weed Technol. 1998 June;12(2):326–36.

[10] Gaskin JF, Schaal BA. Hybrid *Tamarix* widespread in U.S. invasion and undetected in native Asian range. Proc Natl Acad Sci. 2002;99(17):11256–9. 12177412 10.1073/pnas.132403299 PMC123243

[11] Jarnevich CS, Evangelista P, Stohlgren TJ, Morisette J. Improving National-Scale Invasion Maps: Tamarisk in the Western United States. West North Am Nat. 2011;71(2):164–75.

[12] Glenn EP, Morino K, Nagler PL, Murray RS, Pearlstein S, Hultine KR. Roles of saltcedar (Tamarix spp.) and capillary rise in salinizing a non-flooding terrace on a flow-regulated desert river. J Arid Environ. 2012;79:56–65.

[13] Glenn EP, Nagler PL, Morino K, Hultine KR. Phreatophytes under stress: transpiration and stomatal conductance of saltcedar (Tamarix spp.) in a high-salinity environment. Plant Soil. 2013;371(1–2):655–72.

[14] Stromberg JC, Lite SJ, Marler R, Paradzick C, Shafroth PB, Shorrock D, et al. Altered stream-flow regimes and invasive plant species: the *Tamarix* case. Glob Ecol Biogeogr. 2007;16(3):381–93.

[15] Gaskin JF, Kazmer DJ. Introgression between invasive saltcedars (*Tamarix chinensis* and *T. ramosissima*) in the USA. Biol Invasions. 2009;11(5):1121–30.

[16] Gaskin JF. The role of hybridization in facilitating tree invasion. AoB Plants. 2016. plw079. 10.1093/aobpla/plw079 PMC5391693 28028055

[17] Global Biodiversity Information Facility. GBIF Occurrence Download for Tamarix chinensis. 2024.

[18] Global Biodiversity Information Facility. GBIF Occurrence Download for Tamarix ramosissima. 2024.

[19] Fick SE, Hijmans RJ. WorldClim 2: new 1-km spatial resolution climate surfaces for global land areas. Int J Climatol. 2017;37(12):4302–15.

[20] Dinno A. Nonparametric Pairwise Multiple Comparisons in Independent Groups using Dunn’s Test. Stata J Promot Commun Stat Stata. 2015;15(1):292–300.

[21] Schoener TW. Nonsynchronous Spatial Overlap of Lizards in Patchy Habitats. Ecology. 1970;51(3):408–18.

[22] Warren DL, Glor RE, Turelli M, ENVIRONMENTAL NICHE EQUIVALENCY VERSUS. CONSERVATISM: QUANTITATIVE APPROACHES TO NICHE EVOLUTION. Evolution. 2008;62(11):2868–83. 18752605 10.1111/j.1558-5646.2008.00482.x

[23] Broennimann O, Fitzpatrick MC, Pearman PB, Petitpierre B, Pellissier L, Yoccoz NG, et al. Measuring ecological niche overlap from occurrence and spatial environmental data. Glob Ecol Biogeogr. 2012;21(4):481–97.

[24] Di Cola V, Broennimann O, Petitpierre B, Breiner FT, D’Amen M, Randin C, et al. ecospat: an R package to support spatial analyses and modeling of species niches and distributions. Ecography. 2017 June;40(6):774–87.

[25] Phillips SJ, Anderson RP, Schapire RE. Maximum entropy modeling of species geographic distributions. Ecol Model. 2006;190(3–4):231–59.

[26] Elith J, Kearney M, Phillips S. The art of modelling range-shifting species: *The art of modelling range-shifting species*. Methods Ecol Evol. 2010;1(4):330–42.

[27] Morales NS, Fernández IC, Baca-González V. MaxEnt’s parameter configuration and small samples: are we paying attention to recommendations? A systematic review. PeerJ. 2017;5:e3093. 28316894 10.7717/peerj.3093 PMC5354112

[28] Cobos ME, Peterson AT, Barve N, Osorio-Olvera L. kuenm: an R package for detailed development of ecological niche models using Maxent. PeerJ. 2019;7:e6281. 30755826 10.7717/peerj.6281 PMC6368831

[29] Boyce MS, Vernier PR, Nielsen SE, Schmiegelow FKA. Evaluating resource selection functions. Ecol Model. 2002;157(2–3):281–300.

[30] Fitzpatrick MC, Weltzin JF, Sanders NJ, Dunn RR. The biogeography of prediction error: why does the introduced range of the fire ant over-predict its native range? Glob Ecol Biogeogr. 2007;16(1):24–33.

[31] Fielding AH, Bell JF. A review of methods for the assessment of prediction errors in conservation presence/absence models. Environ Conserv. 1997;24(1):38–49.

[32] Hanley JA, McNeil BJ. The meaning and use of the area under a receiver operating characteristic (ROC) curve. Radiology. 1982;143(1):29–36. 7063747 10.1148/radiology.143.1.7063747

[33] Li HQ, Liu XH, Wang JH, Xing LG, Fu YY. Maxent modelling for predicting climate change effects on the potential planting area of tuber mustard in China. J Agric Sci. 2019;157(5):375–81.

[34] Atwater DZ, Ervine C, Barney JN. Climatic niche shifts are common in introduced plants. Nat Ecol Evol. 2017;2(1):34–43. 29203919 10.1038/s41559-017-0396-z

[35] Friedman JM, Roelle JE, Gaskin JF, Pepper AE, Manhart JR. Latitudinal variation in cold hardiness in introduced *Tamarix* and native *Populus*. Evol Appl. 2008;1(4):598–607. 25567800 10.1111/j.1752-4571.2008.00044.x PMC3352386

[36] Pasiecznik N. Tamarix ramosissima (saltcedar) [Internet]. 2007 [cited 2025 May 14]. p. 52503. Available from: http://www.cabidigitallibrary.org/doi/10.1079/cabicompendium.52503

[37] Dougherty LR, Frost F, Maenpaa MI, Rowe M, Cole BJ, Vasudeva R, et al. A systematic map of studies testing the relationship between temperature and animal reproduction. Ecol Solut Evid. 2024;5(1):e12303.

[38] Liu Y, Li J, Zhu Y, Jones A, Rose RJ, Song Y. Heat Stress in Legume Seed Setting: Effects, Causes, and Future Prospects. Front Plant Sci. 2019 July;31:10:938. 10.3389/fpls.2019.00938 PMC6684746 31417579

[39] Zinn KE, Tunc-Ozdemir M, Harper JF. Temperature stress and plant sexual reproduction: uncovering the weakest links. J Exp Bot. 2010;61(7):1959–68. 20351019 10.1093/jxb/erq053 PMC2917059

[40] Jumrani K, Bhatia VS. Interactive effect of temperature and water stress on physiological and biochemical processes in soybean. Physiol Mol Biol Plants. 2019;25(3):667–81. 31168231 10.1007/s12298-019-00657-5 PMC6522612

[41] Sadok W, Lopez JR, Smith KP. Transpiration increases under high-temperature stress: Potential mechanisms, trade‐offs and prospects for crop resilience in a warming world. Plant Cell Environ. 2021 July;44(7):2102–16. 10.1111/pce.13970 33278035

[42] Guo X, Zha T, Jia X, Wu B, Feng W, Xie J, et al. Dynamics of Dew in a Cold Desert-Shrub Ecosystem and Its Abiotic Controls. Atmosphere. 2016;7(3):32.

[43] Ma H, Tian C, Feng G, Yuan J. Ability of multicellular salt glands in Tamarix species to secrete Na + and K+ selectively. Sci China Life Sci. 2011;54(3):282–9. 21416329 10.1007/s11427-011-4145-2

[44] Horton JL, Clark JL. Water table decline alters growth and survival of Salix gooddingii and Tamarix chinensis seedlings. Ecol Manag. 2001;140(2–3):239–47.

[45] Carter JM, Nippert JB. Physiological Responses of Tamarix ramosissima to Extreme NaCl Concentrations. Am J Plant Sci. 2011;02(06):808–15.

[46] Fu A, Li W, Chen Y. The threshold of soil moisture and salinity influencing the growth of Populus euphratica and *Tamarix ramosissima* in the extremely arid region. Environ Earth Sci. 2012;66(8):2519–29.

[47] Hutchinson GE. Concluding Remarks. Cold Spring Harb Symp Quant Biol. 1957;22(0):415–27.

[48] Guisan A, Petitpierre B, Broennimann O, Daehler C, Kueffer C. Unifying niche shift studies: insights from biological invasions. Trends Ecol Evol. 2014;29(5):260–9. 24656621 10.1016/j.tree.2014.02.009

[49] Thompson E. Mesembryanthemum crystallinum (crystalline iceplant) [Internet]. 2015 [cited 2025 May 16]. p. 115578. Available from: http://www.cabidigitallibrary.org/doi/10.1079/cabicompendium.115578

[50] Sheidai M, Koohdar F. Evidence for ancient introgression and gene flow in the genus Tamarix L. (Tamicaceae): a computational approach. Genet Resour Crop Evol. 2023;70(6):1653–61.

[51] Bradley BA, Wilcove DS, Oppenheimer M. Climate change increases risk of plant invasion in the Eastern United States. Biol Invasions. 2010 June;12(6):1855–72.

[52] Bellard C, Jeschke JM, Leroy B, Mace GM. Insights from modeling studies on how climate change affects invasive alien species geography. Ecol Evol. 2018 June;8(11):5688–700. 10.1002/ece3.4098 PMC6010883 29938085

[53] Evans AE, Jarnevich CS, Beaury EM, Engelstad PS, Teich NB, LaRoe JM, et al. Shifting hotspots: Climate change projected to drive contractions and expansions of invasive plant abundance habitats. Divers Distrib. 2024;30(1):41–54.

[54] Valéry L, Fritz H, Lefeuvre JC, Simberloff D. Invasive species can also be native…. Trends Ecol Evol. 2009;24(11):585–585. 19762116 10.1016/j.tree.2009.07.003

[55] Simberloff D, Martin JL, Genovesi P, Maris V, Wardle DA, Aronson J, et al. Impacts of biological invasions: what’s what and the way forward. Trends Ecol Evol. 2013;28(1):58–66. 22889499 10.1016/j.tree.2012.07.013

[56] Lodge DM, Williams S, MacIsaac HJ, Hayes KR, Leung B, Reichard S, et al. BIOLOGICAL INVASIONS: RECOMMENDATIONS FOR U.S. POLICY AND MANAGEMENT. Ecol Appl. 2006;16(6):2035–54. 17205888 10.1890/1051-0761(2006)016[2035:birfup]2.0.co;2

[57] Intergovernmental Panel on Climate Change. Climate Change 2023: Synthesis Report. Contribution of Working Groups I, II and III to the Sixth Assessment Report [Internet]. IPCC; 2023 [cited 2025 May 20]. Available from: https://www.ipcc.ch/report/ar6/syr/

[58] Harsch MA, HilleRisLambers J. Climate Warming and Seasonal Precipitation Change Interact to Limit Species Distribution Shifts across Western North America. Carcaillet C, editor. PLOS ONE. 2016 July 22;11(7):e0159184. 10.1371/journal.pone.0159184 PMC4957754 27447834

[59] Wang D, Xu X, Zhang H, Xi Z, Abbott RJ, Fu J, et al. Abiotic Niche Divergence of Hybrid Species from Their Progenitors. Am Nat. 2022;200(5):634–45. 36260852 10.1086/721372

[60] Hanberry BB, He HS, Dey DC. Sample sizes and model comparison metrics for species distribution models. Ecol Model. 2012;227:29–33.

