# Supplementary material for: Invasion of old world Tamarix chinensis and T. ramosissima in the new world: ecological niche shifts during the invasion process
Source: BMC Plant Biol. 2026 Mar 2;26:548. doi: 10.1186/s12870-026-08415-y (PMC13020398; doi:10.1186/s12870-026-08415-y)
Supplement: Supplementary file 1 — Supplementary Material 1. Supplementary Figure S1: Heatmap of Pearson correlation coefficients among 19 bioclimatic variables used in this study. Colors from blue to red indicate negative and positive correlations, with intensity reflecting correlation strength (−1 ≤ r ≤ 1). Highly correlated variables were identified to guide variable selection for ecological niche modeling and reduce multicollinearity. Supplementary Figure S2: Distribution models of Tamarix chinensis and T. ramosissima calibrated in the invasive range (a, b) and projected to the native range (c, d). Panel (a) shows the distribution model of T. chinensis calibrated in the invasive range, while panel (b) shows the model for T. ramosissima calibrated in the invasive range. Panels (c) and (d) display reciprocal distribution models trained in the native range for T. chinensis and T. ramosissima, respectively. Supplementary Figure S3: Modeled global habitat suitability for the species complex under three climate scenarios: (a) present-day climate, (b) future climate in 2070 under SSP1-2.6, and (c) future climate in 2070 under SSP5-8.5. Areas shown in red indicate the highest predicted habitat suitability. Map boundaries indicate the study area and do not necessarily correspond to officially recognized national borders. Supplementary Table S1: Suitable habitat areas (km²) for Tamarix species and the Species complex under current and future climate scenarios (SSP1-2.6 and SSP5-8.5 for 2070) in Eurasia and North America. [file 12870_2026_8415_MOESM1_ESM.zip › 12870_2026_8415_MOESM1_ESM/Supplementary/Supplementary Table S1.docx]

**Supplementary Table S1** Suitable habitat areas (km²) for *Tamarix* species and the Species complex under current and future climate scenarios.

| Species | Scenario | Native area (km²) | Invasive area (km²) |
| --- | --- | --- | --- |
| *Tamarix chinensis* | Current | 35,899,548.75 | 12,692,155.69 |
|  | SSP1-2.6 (2070) | 35,898,807.47 | 12,065,860.14 |
|  | SSP5-8.5 (2070) | 41,371,446.99 | 16,896,076.15 |
| *T. ramosissima* | Current | 39,980,420.08 | 16,362,690.42 |
|  | SSP1-2.6 (2070) | 37,264,037.40 | 16,498,647.82 |
|  | SSP5-8.5 (2070) | 37,775,180.96 | 18,301,335.39 |
| Species complex | Current | 38,304,635.77 | 14,862,419.12 |
|  | SSP1-2.6 (2070) | 39,750,553.37 | 16,524,329.07 |
|  | SSP5-8.5 (2070) | 42,051,016.65 | 18,713,349.57 |

**Note**: Habitat suitability areas were calculated using binary Maxent predictions, based on species-specific thresholds. Future climate scenarios SSP1-2.6 and SSP5-8.5 refer to projected climate conditions for the year 2070. Suitable habitat areas were measured using the continents of Eurasia and North America as boundaries.
